# Ten year trend analysis of malaria prevalence in Kola Diba, North Gondar, Northwest Ethiopia

**DOI:** 10.1186/1756-3305-5-173

**Published:** 2012-08-14

**Authors:** Abebe Alemu, Dagnachew Muluye, Mikrie Mihret, Meaza Adugna, Melkamu Gebeyaw

**Affiliations:** 1 School of Biomedical and Laboratory Sciences, College of Medicine and Health Sciences, University of Gondar, Gondar, Ethiopia

## Abstract

**Background:**

Malaria is caused by protozoan parasites of the genus *Plasmodium*. It is one of the leading causes of illness and death in the world. It is a major public health problem in Ethiopia. Over the past years, the disease has been consistently reported as the first leading cause of outpatient visits, hospitalization and death in health facilities across the country.

**Methods:**

A retrospective study was conducted to determine the prevalence of malaria from peripheral blood smear examinations from the Kola Diba Health Center of Ethiopia. The case notes of all malaria cases reported between 2002–2011 were carefully reviewed and analyzed. Additionally, any malaria intervention activities that had been taken to control malaria were collected using a well-prepared checklist from the study area.

**Results:**

Within the last decade (2002–2011) a total of 59, 208 blood films were requested for malaria diagnosis in Kola Diba health center and 23,473 (39.6%) microscopically confirmed malaria cases were reported in the town with a fluctuating trend. Regarding the identified *plasmodium* species, *Plasmodium falciparum* and *Plasmodium vivax* accounted for 75% and 25% of malaria morbidity, respectively. Malaria was reported in all age groups and both sexes, but the 15–44 year age group and males were more affected. Despite the apparent fluctuation of malaria trends in the area, the highest peak of malaria cases was reported during spring seasons.

**Conclusion:**

Comparatively, after the introduction of the current malaria control strategies, the morbidity and mortality by malaria is decreasing but malaria is still a major health problem and the deadly species *P. falciparium* is predominant. Therefore, control activities should be continued in a strengthened manner in the study area considering both *P. falciparium* and *P. vivax.*

## Background

Malaria is a protozoan disease caused by parasites of the genus *Plasmodium*. It is one of the leading causes of illness and death in the world. It is the leading cause of death in children under the age of 5 years and pregnant women in developing countries [1,2]. In 2010 there were an estimated 216 million cases of malaria worldwide, of which 91% were due to *P. falciparum*. The vast majority of cases (81%) were in the African Region followed by South-East Asia (13%) and Eastern Mediterranean Regions (5%) [3]. The disease remains one of the most important causes of human morbidity and mortality with enormous medical, economic and emotional impact in the world. In most African countries including Ethiopia, the number of cases reported annually fell by at least a quarter and, in some instances, by more than a half, between 2000 and 2010 [3].

Despite considerable progress in malaria control over the past decade, it is the most important public health problem in Ethiopia where an estimated 68% of the population lives in malarious areas and three quarters of the total land mass is regarded as malarious [4]. *Plasmodium falciparum* and *Plasmodium vivax* are the two predominant malaria parasites, distributed all over the country and accounting for 60% and 40% of malaria cases, respectively. Reports indicate that clinical malaria accounts for 10%-40% of all out patient consultations, with corresponding proportional morbidity among children under 5 years in age being 10% - 20% [5,6].

Prevention and control activities of malaria in Ethiopia are implemented as guided by the National Strategic Plan to ultimately reduce the burden of malaria to a level where it is no longer a public health problem. Four major intervention strategies that are being applied in Ethiopia to combat malaria were: early diagnosis and prompt treatment, selective vector control that involves use of indoor residual spraying (IRS), insecticide-treated mosquito nets (ITNs) and environmental management [7]. A major challenge for malaria epidemiologists is to evaluate the strengths and weaknesses of both methods in estimating malaria incidence and time trends, especially as malaria control programmes are intensified worldwide [8].

Regardless of decades of sustained control efforts, malaria still remains as the major cause of morbidity, mortality and socioeconomic problems in Ethiopia because malaria control is a big challenge due to many factors. The complexity of the disease control process, expensiveness of the control program, resistance of the parasite to antimalarial drugs and vectors to insecticides are some of the challenges [9-14]. Moreover, currently in Ethiopia different reports indicate that malaria is decreasing but the exact factors for its reduction are not well defined. Therefore, this study was initiated to analyse the ten year trend prevalence of malaria and to assess the impact of the current national malaria control activities on malaria prevalence in the study area. The study will provide current trends of malaria and envisages that it might strengthen the information so far for scaling up and to design effective communication strategies to combat malaria in the study area.

## Methods

### Study area

The study was conducted at Kola Diba health center (first health center in Ethiopia), Dembia District, which is located in north Gondar, Amhara region, 729 km north of Addis Ababa. Dembia is one of the districts of North Gondar Administrative Zone, known for its flat land. This district is malarious and covers an area of 1270 km with a total population of about 263000 and the population of the district is predominately Amhara, Orthodox Christianity being the main religion. The altitude of the district ranges between 1750 and 2100 m above sea level. The District of Dembia lies close to Lake Tana (the largest lake in Ethiopia) and the majority of the population depends on subsistence farming. The administrative centre Kola Diba is only 35 km from the ancient city of Gondar. Malaria is the most prevalent seasonal disease in the area, accounted as second of all the reported diseases in the health center and October to December is the peak malaria transmission season in the area. Both *P.vivax* and *P.falciparum* exist in the area with *P.falciparum* prevailing all year.

### Study design

A retrospective study was conducted to determine the ten year trend prevalence of malaria by reviewing blood film malaria reports at Kola Diba health center.

### Data collection

#### Malaria data

Ten year malaria prevalence data was obtained from Kola Diba heath center. In this health center, peripheral smear examination of a well-prepared and well-stained blood film is used as the gold standard in confirming the presence of the malaria parasite as WHO protocol. In Ethiopia, the staining techniques and blood film examination for malaria parasite detection were conducted according to a standard operating procedure (SOP) in each health center throughout the country. Therefore, for this study purpose we have collected ten year (2002–2011) malaria data at Kola Diba health center from December – February 2011.

#### Factors affecting malaria trends

During malaria data collection, any malaria intervention activities that had been taken in each year to control malaria were collected using a well-prepared checklist from different responsible offices/agencies or individuals. There was increased attention to malaria control and preventive activities by different responsible bodies, increased awareness of the community on use of insecticide treated bed nets (ITNs) and other malaria control activities through health education, increased accessibility of ITNs to community, increment of budget for malaria control and prevention activities (personal communication with head of health center). But in the study area there were no special factors that attributed the increased or decreased occurrence of malaria cases.

Other factors such as antimalarial drug resistance of the study area were obtained from Hinari and Enterz- PubMed web site and through personal communication. In the study area, chloroquine was the most abundant and commonly used antimalarial drug until resistance was reported. In Ethiopia, the increased resistance of *P. falciparum* to chloroquine (CQ) and sulfadoxine pyrimethamine (SP) necessitated a change as first-line antimalarial drug for the treatment of *P. falciparum*. Consequently, Artemether/Lumefantrine (Coartem®) (AL) was adopted in 2004 but it became available in all regions of the country in 2005 [15-17]. Currently the common drug used for treatment of *P. vivax* malaria in the study area and throughout the country is chloroquine, because data on chloroquine resistance to *P.vivax* malaria is not sufficient enough to warrant a change. Again, in the study area both *P.falciparum* and *P.vivax* are common plasmodium species and self treatment is also common, so there is a danger that parasites have developed or will develop the resistance to Coartem®, chloroquine, respectively.

#### Data analysis

Data was entered and analyzed by SPSS 16 software package. The frequency distribution of both dependent and independent variables were worked out by using crosstab. Finally, the data was described and presented using figures.

#### Ethical consideration

This ten year data was collected after ethical clearance obtained from The School of Biomedical and Laboratory Sciences, College of Medicine and Health Science, University of Gondar. After discussing the purpose and method of the study, written permission was sought from the Head of Kola Diba health centre before the data collection.

## Results

### Annual trends of malaria prevalence in Kola Diba health center

**Figure 1:**
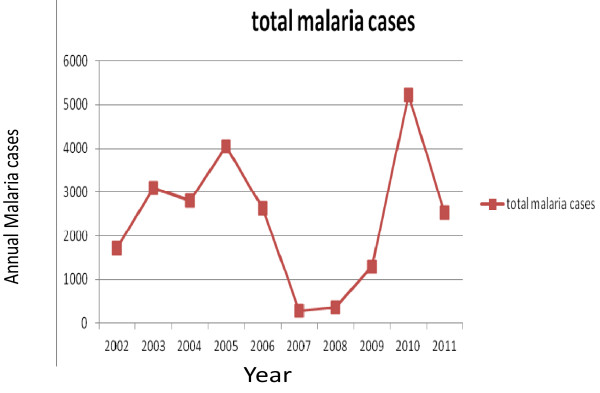
Annual trends in total malaria cases in Kola Diba health center from 2002–2011.

Within the last decade (2002–2011) a total of 59, 208 blood films were requested for malaria diagnosis in Kola Diba health center and 23,473 (39.6%) microscopically confirmed malaria cases were reported in the town with mean malaria cases of 2, 347.3. There was a fluctuating trend of malaria within the last decade, with the minimum (285) number of microscopically confirmed malaria cases being reported in 2007 and the maximum (5201) microscopically confirmed cases of malaria being reported in 2002 (Figure 1).

**Figure 2:**
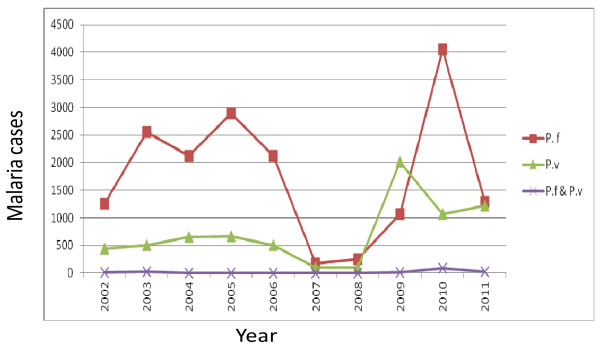
Species trends of malaria parasites in Kola Diba health center from 2002–2011.

Regarding the identified plasmodium species, both species of plasmodium were reported in each year with *Plasmodium falciparium* being the predominant species in the study area and *Plasmodium falciparum* and *Plasmodium vivax* accounted for 75% and 25% of malaria morbidity, respectively in the area. Recently, in the year 2010 to 2011 *P. falciparium* was decreasing but *P. vivax* was increasing, which shows that there was a trend shift from *P. falciparium* to *P. vivax* in the study area. Generally, mixed infection is insignificant in the area (0.3%), (Figure 2).

### Prevalence of malaria parasites in relation to sex and age in Kola Diba health center

**Figure 3:**
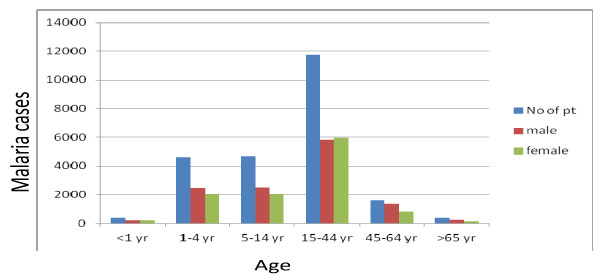
The prevalence of malaria parasites by sex and age in Kola Diba health centre from 2002–2011.

According to our record review in the last decade in the study area, males were more affected than females by malaria parasites but vary year to year. The infection rates among males were 12, 358 (52.6%) and females were 11, 115 (47.3%). Malaria was reported in all age groups in the area but the age group of 15–44 years were more affected, with a prevalence rate of 11769 (50.1%), followed by 5–14 year olds and 1–4 year olds with the prevalence rate 4717 (20%) and 4599 (19.6%), respectively. Except for the 15–44 age group, females were more affected than males in the study area (Figure 3).

**Figure 4:**
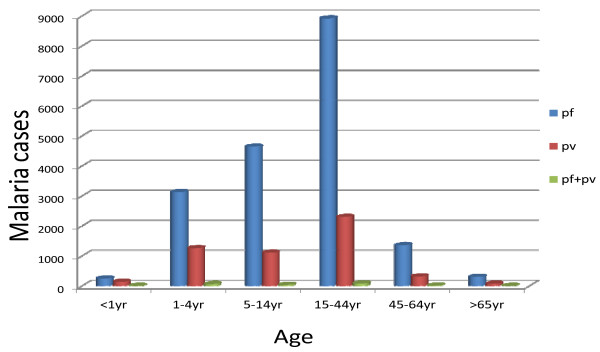
**The distribution of *Plasmodium* in different age groups in the Kola Diba health center from 2002–2011.**

In relation to *Plasmodium* species and age groups in the study area *P.falciparum* was the predominant parasite in all age groups and it was higher in the 15–44 age group and 5–14 year age group with a prevalence rate of 9204(78.2%) and 3575(75.7%), respectively. The 15–44 age group was more affected by *P.vivax* 2485 (21.1%) followed by 1–4 year olds,1371 (29.8%) and 5–14 year 1109 (23.5%) whereas mixed infection was the least prevalent in all age groups (Figure 4).

### Seasonal variation of malaria prevalence in the Kola Diba health center

**Figure 5:**
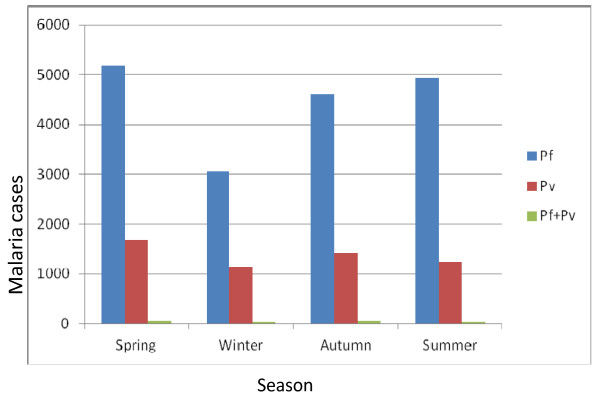
**The distribution of *Plasmodium* in different seasons in Kola Diba health from 2002–2011.**

Despite the apparent fluctuation of malaria trends in the study area, malaria cases occurred in almost every month and season of the year. The highest peak of malaria cases in almost all years was observed during spring (September, October and November) and the minimum malaria cases were observed during winter (December, January and February) seasons. At species level, the maximum number of cases of *P.falciparum* were observed in spring and summer, but the maximum number of cases of *P.vivax* were observed in spring, followed by autumn (March, April and May) and in both species the minimum number of malaria cases were observed during winter (December, January, February) (Figure 5).

## Discussion

Malaria is a huge public health problem in terms of morbidity and burden on health care facilities, accounting for the increasing percentage of outpatient consultations in most health facilities in different regions in Ethiopia [12]. The present study revealed that the burden of malaria was high in the study area where the most deadly species, *P. falciparum*, accounted for 75%. This is lower than other studies reported from Ethiopia [18] and Eretria [19], but it is higher compared to a retrospective study done in Malaysia [20]. This difference may be due to the type of study design used, climatological differences, altitude variation, malaria diagnosis technique variation, skill of the laboratory personnel to detect and identify malaria parasites and other factors that affect malaria case occurrences in different study areas.

The results of our study revealed that during the last ten years, a fluctuating trend occurrence of malaria cases was observed in the study area. A decrease in the number of malaria cases occurred from 2005–2008 with a minimum number of malaria cases reported in 2007. However, there was an increase in the number of malaria cases from 2008–2010 with the peak number of malaria cases being reported most recently in 2010. In every year studied the remarkable increment of total malaria cases was mainly due to an increase of *P. falciparum* with little increase of *P.vivax,* which indicates that the deadly plasmodium species is common in the study area.

The reduction of malaria cases from 2004 coincides with the increased availability of the new effective drug Coartem for the treatment of *P. falciparum* malaria at national and local levels [6]. Other possible reasons for malaria reduction during this period (2005–2008) might be due to the increased attention to malaria control and preventive activities by different responsible bodies, increased awareness of the community on use of ITNs, increment of budget for malaria control and prevention activities (personal communication) and climate change at national and international level.

Because the year 2010 was the peak period of malaria case occurrence, which seems to be epidemic and used to occur in a cyclic nature over a five to six year time period. Although the observed decline in malaria could be strongly attributed to the interventions in the subsequent years, under normal conditions the malaria situation could decline independently. There should be a mechanism whereby we can determine that the decline is due to the intervention or other factors such as climate. For example, if the epidemic does not occur over a longer time than the normal epidemic cycle. Observed results in 2011 of increased malaria case reports in the study area coincides with other reports in Ethiopia, which indicated that the usual 5 to 7 year cyclic period has currently changed and we are seeing malaria epidemics every one or two years and also in different places [8].

*Plasmodium falciparium* was the predominant species in the study area and accounted for 75% of malaria morbidity. This finding coincides with the malaria parasite distribution in Ethiopia which indicates that *Plasmodium falciparum* and *Plasmodium vivax* are the two predominant malaria parasites, distributed all over the country and accounting for 60% and 40% of malaria cases, respectively. This study also shows that currently since 2011 *P. falciparium* is decreasing but *P. vivax* is slightly increasing, which indicates a trend shift. This trend shift is similar to other recent study reports in Ethiopia carried out by Jimma [12]. The possible reason for this trend shift from *P. falciparium* to *P. vivax* might be due to the public health importance of *P. vivax* that is frequently overlooked and left in the shadow of the enormous problem caused by *Plasmodium falciparum*[21,22]. In addition, the prevention and control activities of malaria as guided by the National Strategic Plan (2006–2010) mainly focus on *P. falciparium* because it is assumed to be more prevalent and fatal malaria in the country Ethiopia. Other possible reasons might be climate variability and *P. vivax* might have developed resistance for the currently used drug (chloroquine) [11].

Regarding the age groups, 15–44 years (50%) were highly affected age groups followed by 5–14 year olds (19.9%) and 1–4 year olds (19.5%) but less than one (<1) year and greater than and equal to sixty five (≥65) year age groups have less association in parasite infection. *P. falciparum* was more prevalent in the 15–44 age group. The prevalence of malaria parasites among males (53.7%) was higher than females (47.5%). The reason why malaria affected productive age groups (15–44) and more males might be due to the fact that in Dembia agriculture is the main occupation and the area is hot so staying outside the home and sleeping under trees is common during the night time. Due to these and other different reasons the age groups and males are more exposed to anopheles mosquito bites, which can transmit malaria parasites.

Seasonality and year played a role in the transmission of malaria in the study area. The highest peak of malaria cases in almost all year groups was observed during spring (September, October and November). This seasonal occurrence indicates the real malaria transmission for the country Ethiopia. In Ethiopia, the major transmission of malaria follows the June to September rains and occurs between September to December, while the minor transmission season occurs between April to May following the February to March rains. Some localities also experience perennial malaria, because the environmental and climatological situations permit the continual breeding of vectors in permanent breeding sites [6,7].

In general, there was a fluctuation in malaria cases during the last ten years. Many factors might be responsible for seasonal changes, e.g., climatic variables, ecologic and environmental factors, host and vector characteristics, and social and economic determinants such as change in health care infrastructure. Social, biological and economic factors such as mosquito control measures, population immunity, local ecological environment, governmental policy, availability of health facilitates and drug resistance also have an impact on malaria prevalence. Although there were different malaria control activities in each year, such as insecticide spraying, elimination of mosquito breeding sites, health education about malaria, distribution of ITNs and some malaria drugs and other activities to decrease mortality and morbidity of malaria, the prevalence is still sustained. The limitation of this study was lack of availability of some factors that may contribute to the malaria case occurrence in the study area.

## Conclusions

Comparatively, after introduction of current malaria control strategies, the morbidity and mortality of malaria is decreasing, but malaria is still a major health problem and the deadly species *P. falciparium* was most predominant. The highest peak of malaria cases in almost all year groups was observed during the spring seasons. Therefore, control activities should be continued in a strengthened manner in the study area considering both *P. falciparium* and *P. vivax* and the seasonal occurrence of malaria

## Competing interests

Abebe Alemu, Dagnachew Muluye, Mikrie Mihret, Meaza Adugna, Melkamu Gebeyaw declare that they have no competing interests.

## Authors’ contributions

AA: conceived the study, undertook statistical analysis and drafted the manuscript. DM: initiated the study, undertook statistical analysis and has major contribution in drafting the manuscript. MM, MA and MG: initiated the study and made major contributions to the study design and statistical analysis. All authors contributed to the writing of the manuscript and approved the submitted version of the manuscript.
